# Fingolimod Affects Transcription of Genes Encoding Enzymes of Ceramide Metabolism in Animal Model of Alzheimer’s Disease

**DOI:** 10.1007/s12035-020-01908-3

**Published:** 2020-04-30

**Authors:** Henryk Jęśko, Przemysław L. Wencel, Sylwia Wójtowicz, Joanna Strosznajder, Walter J. Lukiw, Robert P. Strosznajder

**Affiliations:** 1 Department of Cellular Signalling, Mossakowski Medical Research Centre, Polish Academy of Sciences, 5 Pawinskiego St., PL-02106 Warsaw, Poland; 2 Laboratory of Preclinical Research and Environmental Agents, Mossakowski Medical Research Centre, Polish Academy of Sciences, 5 Pawinskiego St., PL-02106 Warsaw, Poland; 3 LSU Neuroscience Center, Louisiana State University School of Medicine, New Orleans, LA USA; 4 Department of Neurology, Louisiana State University School of Medicine, New Orleans, LA USA; 5 Department of Ophthalmology, Louisiana State University School of Medicine, New Orleans, LA USA

**Keywords:** Alzheimer’s disease, Ceramide, Fingolimod, Sphingolipid, Sphingosine-1-phosphate, Sphingomyelin synthase

## Abstract

The imbalance in sphingolipid signaling may be critically linked to the upstream events in the neurodegenerative cascade of Alzheimer’s disease (AD). We analyzed the influence of mutant (V717I) amyloid β precursor protein (AβPP) transgene on sphingolipid metabolism enzymes in mouse hippocampus. At 3 months of age AβPP/Aβ presence upregulated enzymes of ceramide turnover on the *salvage pathway*: ceramide synthases (*CERS2*, *CERS4*, *CERS6*) and also ceramidase *ACER3*. At 6 months, only *CERS6* was elevated, and no ceramide synthase was increased at 12 months. However, sphingomyelin synthases, which utilize ceramide on the *sphingomyelinase pathway*, were reduced (*SGMS1* at 12 and *SGMS2* at 6 months). mRNAs for sphingomyelin synthases SGMS1 and SGMS2 were also significantly downregulated in human AD hippocampus and neocortex when compared with age-matched controls. Our findings suggest early-phase deregulation of sphingolipid homeostasis in favor of ceramide signaling. Fingolimod (FTY720), a modulator of sphingosine-1-phosphate receptors countered the AβPP-dependent upregulation of hippocampal ceramide synthase *CERS2* at 3 months. Moreover, at 12 months, FTY720 increased enzymes of ceramide-sphingosine turnover: *CERS4*, *ASAH1*, and *ACER3*. We also observed influence of fingolimod on the expression of the *sphingomyelinase pathway* enzymes. FTY720 counteracted the AβPP-linked reduction of sphingomyelin synthases SGMS1/2 (at 12 and 6 months, respectively) and led to elevation of sphingomyelinase SMPD2 (at 6 and 12 months). Therefore, our results demonstrate potentially beneficial, age-specific effects of fingolimod on transcription of sphingolipid metabolism enzymes in an animal model of AD.

## Introduction

Alzheimer’s disease (AD) is the most widespread neurodegenerative disorder accounting for up to 70% of the estimated 47 million dementia cases present worldwide [1]. Extracellular senile plaques of amyloid β (Aβ) are a hallmark and a crucial element of AD neuropathology [2]. The V717I “London” mutation of Aβ precursor protein (AβPP) has been reported in familial (FAD)/early-onset AD [3]. The mutation increases Aβ production and shifts the AβPP metabolism in favor of the highly neurotoxic Aβ_42_ isoform [4].

**Fig. 1:**
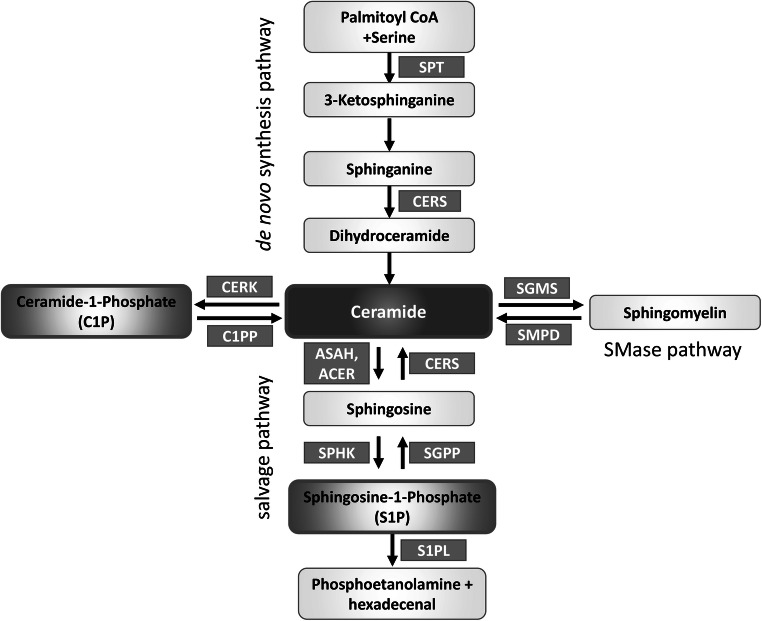
Ceramide biosynthesis pathways. Ceramide (Cer) can be produced on each of the three marked pathways. Serine palmitoyltransferase (SPT) catalyzes the first step in the de novo pathway and controls the rate of ceramide biosynthesis. Ceramide synthases (CERS) take part both in the salvage and de novo pathways, using different substrates (sphingosine and sphinganine). Metabolic shuttling between ceramide and sphingomyelin is done by sphingomyelin synthase—S(G)MS, and sphingomyelin phosphodiesterase (SMPD). Sphingosine is produced from ceramide by ceramidases (ASAH, ACER). Sphingosine can be phosphorylated by the sphingosine kinases (SPHK1 and SPHK2) to form sphingosine-1-phosphate (S1P), while the ceramide kinase CERK phosphorylates ceramide to ceramide-1-phosphate (C1P); both phosphates have signaling activities approximately opposite to those of sphingosine and ceramide. Dephosphorylation of S1P and C1P is carried out by their respective phosphatases, SGPP and C1PP. The highly controlled enzymatic conversion that yields compounds of radically different activities in a single reaction is reflected in the idea of the *sphingolipid rheostat*

Aging of the central nervous system (CNS) creates vulnerable background for the development of the disease and alters numerous signaling and metabolic pathways linked to neuronal phenotype, function, and survival/death. Both aging and AD are accompanied by alterations in the metabolism of bioactive sphingolipids, a precisely regulated network of compounds (Fig. 1) with strong pro- or anti-apoptotic activities [5–8]. The pro-apoptotic sphingolipid ceramide is synthesized on several distinct metabolic pathways and can be converted by ceramidases into sphingosine [9]. A single phosphorylation reaction turns each of them into molecules that typically exert strong pro-survival influence (C1P, ceramide-1-phosphate, and S1P, sphingosine-1-phosphate). S1P modulates cell survival, and also proliferation, differentiation, morphology, neurotransmission, and synaptic plasticity. In addition to its second messenger role, S1P can signal via cell membrane receptors (S1PR1 to S1PR5) that bind G_12/13_, G_q_, and G_i_ proteins [9]. The PI3K-Akt pathway responds to G proteins and modulates transcription factors (TFs) including AP-1 (activator protein-1, which is in turn one of crucial regulators of sphingolipid metabolism enzymes including *SPHK1*, *CERS4*, and *CERS5* [10, 11]), or NF-κB (nuclear factor κB, a pleiotropic sensor of stress and metabolic signals engaged in survival/death decisions and immune activation [12]). The contrasting roles of closely related sphingolipids require precise regulation, as reflected in the term *sphingolipid rheostat*.

Aβ has been shown to alter the expression of genes engaged in sphingolipid signaling [13]. The effect seems to have profound significance in actual AD cases, where early, widespread disturbances in the ceramide-linked metabolic pathways correlate with the disease progression. Ceramide may act as a pro-apoptotic molecule, and—together with deregulated S1P—can also alter stress signaling, AβPP processing, or tau phosphorylation [14]. Ceramide and S1P are engaged in structural aspects of biological membranes, neuronal axons, and synapses (lipid rafts, protein anchoring), and in mitochondrial maintenance [15–20]. Their interactions with the PI3K-Akt and NF-κB pathways allow cross-talk with metabolic control/nutrient sensing [14]. Human AD brain samples display high mRNA levels of *CERS1* and *CERS2* (ceramide synthases), *SPTLC2* (serine palmitoyltransferase), or *SGPL1* (S1P lyase), and low expression of *CERK* (ceramide kinase) or *ASAH1* (acid ceramidase) [8]. These changes result in increased concentrations of some ceramide species and reduced S1P, potentially resulting in a pro-apoptotic shift in the signaling equilibrium [6, 8]. Moreover, elevated ceramides may alter AβPP maturation and its cleavage by secretases, closing the circle of self-fueling pathology [21–23]. Sphingolipid alterations occur very early during disease development, suggesting close links with upstream events of AD pathogenesis [6–8]; moreover, altered sphingolipid content in the cerebrospinal fluid (CSF)/blood may be a useful marker [24, 25].

Although experimental AD models are being criticized for recreating only selected aspects of the disease with varying fidelity [26], they may be highly useful in the elucidation of molecular/biochemical events mediating the neurotoxicity of Aβ [27]. Aβ peptides modulate the enzymes of sphingolipid metabolism and S1P receptors in cellular models; thus, Aβ’s effect appears to be relatively direct [13]. We have recently demonstrated the influence of neuronal expression of V717I AβPP on the mRNA levels of sphingosine kinases and cell surface S1P receptors [27].

Unfortunately, up until now, no therapeutic strategy exists that would protect neuronal cells against degeneration and death in AD. Many types of drugs were developed de novo but without success [26, 28]. Repurposing of already developed and approved compounds is an innovative approach that avoids part of the enormous workload and time required [29]. Fingolimod (FTY720, Gilenya™) is a clinically available compound approved by the U.S. Food and Drug Administration and European Medicines Agency for the treatment of relapsing remitting multiple sclerosis (MS). FTY720 can be administered in multiple ways (orally for the therapy of MS) and crosses the blood-brain barrier [30]. FTY720 is a sphingosine analogue; its phosphorylation by sphingosine kinases (SphKs) changes it into an analogue of S1P, capable of binding the cell surface S1P receptors. The mechanism of action employed in its current therapeutic application comprises of activation-induced feedback internalization of cell surface S1P receptors, and the resulting inhibition of S1P signaling in immune cells. Binding of S1P or FTY720P (phosphorylated, active form of fingolimod) to S1PRs activates the PI3K-Akt pathway, whose disruption in AD [31] may be one of pathomechanisms of neurodegeneration [32, 33]. Through PI3K-Akt, S1P receptors can inhibit GSK-3β, a tau kinase [34]. S1PRs also modulate p38, ERK, Jnk, and the TNF receptor–associated factor TRAF2. Through modulation of cellular signaling, S1P and S1PRs target nuclear transcription factors, including AP-1, NF-κB, or FOXO3a [17, 31, 35, 36], which influence pathways important for stress and immune response [10, 11, 36, 37], and also ensure feedback regulation of selected sphingolipid metabolism enzymes and S1P receptors. The S1PR-sensitive changes in gene expression might also alter axonal connectivity [38] and the availability of neurotrophic factors [39]. Therefore, at least majority of these TFs should be available for pharmaceutical modulation with fingolimod, enabling modulation of critical aspects of the neurodegenerative process. S1P and FTY720P inhibit class I histone deacetylases (HDACs) [40–42]. Interaction with HDACs may have direct consequences in neurodegenerative and related conditions through modification of activated microglia phenotypes [43, 44]. FTY720 shows protective activity in demyelination and blood-brain barrier (BBB) damage [44–46]. Moreover, fingolimod has also been suggested to exhibit (possibly selective) antibacterial properties, which might contribute to its efficiency in BBB and neuronal protection [47]. Following the known fact of intestinal dysfunction in PD, the potential role of gut microbiota alterations (dysbiosis) and the influence of FTY720 have also been analyzed in the disease [48]. Finally, FTY720 enhances learning/memory while reduction of S1P levels impairs memory through high HDAC activity and altered gene expression profile [41]. Therefore, FTY720 constitutes an attractive potential drug candidate, although its complex influence upon neuronal phenotype and survival must be characterized in depth to ensure any successful therapeutic usage in neurodegenerative disorders.

The aim of the study was to analyze the effect of the administration of the potentially neuroprotective, clinically available drug FTY720 on the *AβPP* (V717I)-induced changes in the expression of genes coding enzymes engaged in ceramide metabolism in the hippocampus of 3-, 6-, and 12-month old mice.

## Materials and Methods

### Animals

Female FVB-Tg(Thy1; APP LD2/B6) mice express human AβPP harboring the “London” V717I mutation under control of thy1 promoter fragment which displays specificity towards neurons of the brain and spinal cord [27, 49, 50]. The model successfully recreates a relatively broad spectrum of behavioral, electrophysiological, and biochemical features of AD [49, 50]. The mice start displaying first behavioral abnormalities up to 8 weeks of age [49]. Starting from the age of approximately 3 months, the animals gradually develop agitation; cognitive disturbances appear at 3 to 6 months. These changes are accompanied by altered reactivity to neurotransmitters (observed at 3–4 months of age) and electrophysiological alterations (at between 5 and 7 months) [49]. Sizeable cortical and hippocampal Aβ histopathology was observed starting from the age of 13 months [49, 50].

The AβPP-expressing animals were used at the age of 3, 6, or 12 months to compare gene expression patterns with controls, which did not inherit the transgene. Animals were treated intraperitoneally, daily for 2 weeks (15 injections) with 1 mg/kg b.w. FTY720 in 0.9% NaCl (or NaCl only for control treatment), as described previously [27]. The dose and duration chosen were based on analysis of literature data [51–53] and were identical as used in our previous work where we observed an array of relatively mild changes in response to the treatment [27]. One day after the last dose, the mice were decapitated and their brains isolated on ice, dissected, and flash-frozen in liquid nitrogen.

The Animal House of the Mossakowski Medical Research Centre PAS, Warsaw, Poland, bred the mice under specific pathogen-free (SPF) conditions in controlled temperature/humidity conditions, 12-h light/dark cycle. Every effort was made to minimize the number of animals and to reduce the amount of pain, distress, and/or discomfort. All experiments were approved by the IV Local Ethics Committee for Animal Experimentation in Warsaw and performed in accordance with guidelines of the EU Directive 2010/63/EU and with the ARRIVE guidelines.

### Real-time Polymerase Chain Reaction Measurement of Gene Expression

The measurements were performed as described previously [27]. Briefly, nucleic acids were extracted using the Chomczynski method with TRI reagent (Sigma-Aldrich/Merck), DNA digested with DNase I (Sigma-Aldrich), and the concentration and purity of obtained RNA assessed spectrophotometrically (A_260_/A_280_). Total RNA reverse transcription was performed with AMV (avian myeloblastosis virus) reverse transcriptase and random primers (Applied Biosystems, Foster City, CA, USA). Real-time PCR was done using TaqMan Gene Expression Assays on Applied Biosystems 7500 Real-Time PCR System. The following genes were analyzed: *ACER2* (Mm00519876_m1), *ACER3* (Mm00502940_m1), *ASAH1* (Mm00480021_m1), *CERS2* (Mm01258345_g1), *CERS4* (Mm00482658_m1), *CERS6* (Mm00556165_m1), *SGMS1* (Mm00522643_m1), *SGMS2* (Mm00512327_m1), *SMPD2* (Mm01188195_g1), *SPTLC1* (Mm00447343_m1), and *SPTLC2* (Mm00448871_m1). Gene expression was calculated using the ddCt method and normalized against actin beta (*ACTB*, Mm00607939_s1).

### Statistical Analysis

mRNA expression levels are expressed as Rq (relative quantity). Each value is a mean ± SEM of three to five samples in tri- to quadruplicate (*n* = 3 to 5). ANOVA (two-way analysis of variance) with Tukey post hoc test was used for multiple comparisons. Statistical significance was accepted at *p* < 0.05. The analysis was performed using GraphPad Prism package (GraphPad Software, San Diego, CA).

### Control and AD Brain Tissue Analysis

Human brain samples were selected from archived tissues or extracts at the Louisiana State University Neuroscience Center, New Orleans, LA, from the University of California at Irvine CA, USA, and from the Oregon Health Sciences Center, Portland, OR, USA; archived RNA samples were also obtained from the University of Toronto (Toronto, Canada). All RNAs were isolated from sporadic AD and control hippocampal and superior temporal lobe neocortical tissue samples having a mean post-mortem interval (PMI; death to brain-freezing interval) of 4.2 h or less as PMI is a factor that can affect RNA quality [54–56]. “Center to establish a registry for Alzheimer’s disease-National Institutes of Health” (CERAD-NIH) criteria were used to categorize AD tissues in accordance with established guidelines; AD tissues used had a clinical dementia rating (CDR; an index of cognitive decline) ranging from 1 to 3, indicating mild to a severe stage [55, 56]. Brain tissues were used in accordance with the institutional review board/ethical guidelines at the Louisiana State University Health Sciences Center and donor institutions [54–56].

### Microarray-Based Analysis of Gene Expression Using DNA Arrays

Total RNA was isolated from control and AD-affected human brain neocortex and hippocampus using TRIzol (Invitrogen) as previously described [54–59]; RNA quality was assessed using an Agilent Bioanalyzer 2100 (Lucent Technologies/Caliper Technologies; Palo Alto, CA, USA) and RNA integrity number (RIN) values were typically 8.0–9.0 indicating high-quality total RNA [60–62]. Control and AD brain RNA samples were labelled, hybridized, and analyzed using 27,000 mRNA target GeneChips (Affymetrix, Palo Alto, CA, USA) as previously described [54, 55, 60–62].

## Results

**Fig. 2:**
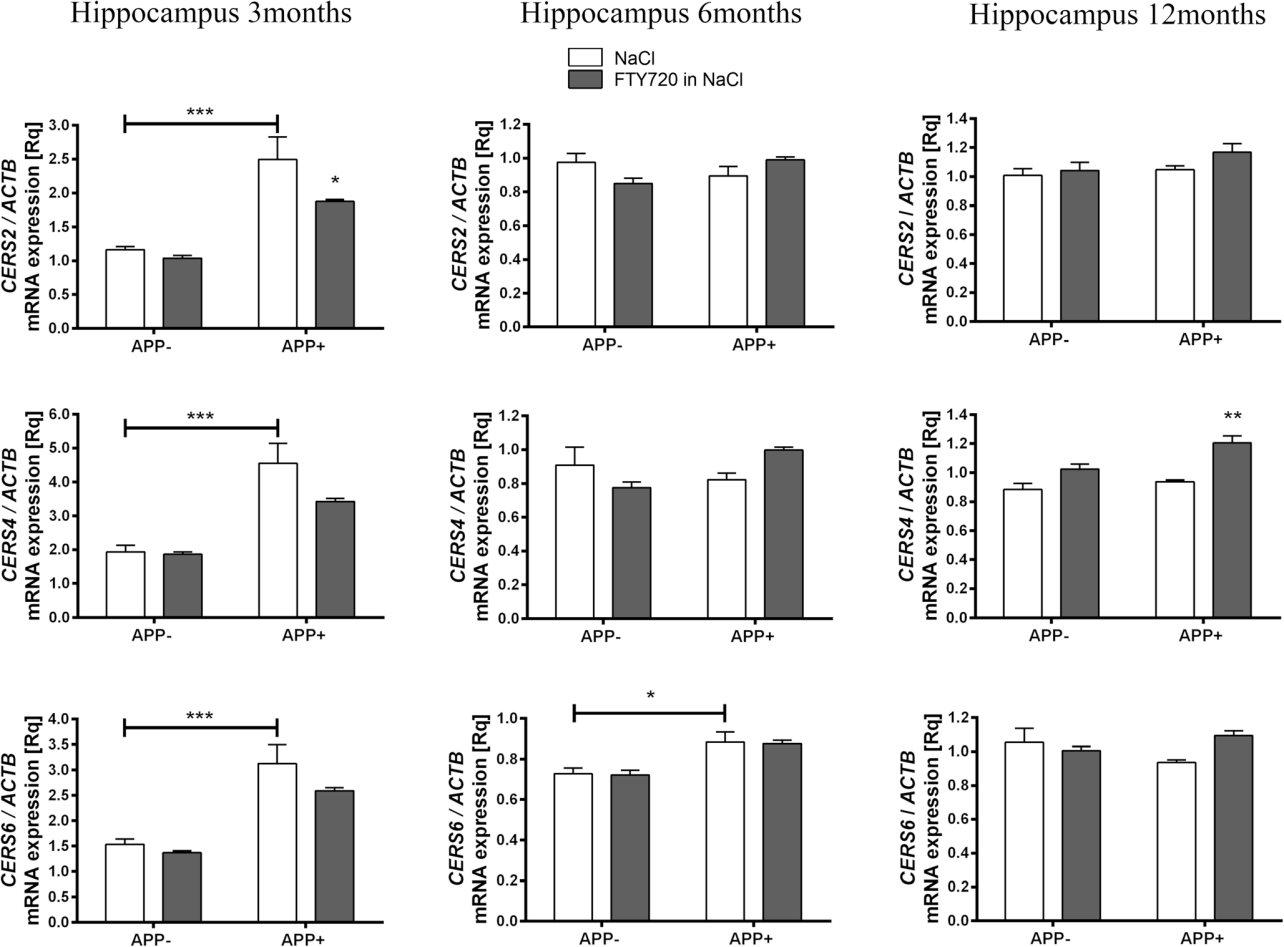
The effect of fingolimod (FTY720) on mRNA expression of ceramide synthases in the hippocampus of *AβPP* (V717I)-transgenic mouse. mRNA expression of *CERS2*, *CERS4*, and *CERS6* was measured with real-time PCR in the hippocampus of *AβPP*-transgenic and control mice at the age of 3, 6, and 12 months. *p < 0.05; **p < 0.01; ***p < 0.001 as compared with the corresponding controls (horizontal bar: APP^−^ mock-treated with NaCl vs. NaCl-administered APP^+^ mice; stars with no horizontal bar mean significant difference between NaCl- and FTY720-treated animals within each group)—ANOVA with Tukey post hoc test

The expression of the disease-related (V717I) variant of human *AβPP* has exerted an age-specific effect on numerous genes linked to ceramide metabolism in the hippocampus. Strikingly, we observed most changes in the youngest age group. The expression of serine palmitoyltransferase (*SPTLC*), the rate-limiting enzyme of the de novo ceramide synthesis did not change in the hippocampus of 3, 6, or 12 months old animals in response to AβPP expression (data not shown). Alterations in the activity of the genes coding other enzymes of ceramide metabolism may have important influence on the delicate signaling equilibrium. In the hippocampus of 3-month-old transgenic mice (APP^+^), ceramide synthases *CERS2*, *CERS4*, and *CERS*6 (which take part both in the de novo and *salvage* pathways**)** were significantly upregulated as compared with control animals (Fig. 2). However, the effect gradually disappeared at later ages. Among ceramide synthases, only *CERS6* was elevated at 6 months, while no *CERS* gene expression was altered by AβPP at 12 months (Fig. 2).

**Fig. 3:**
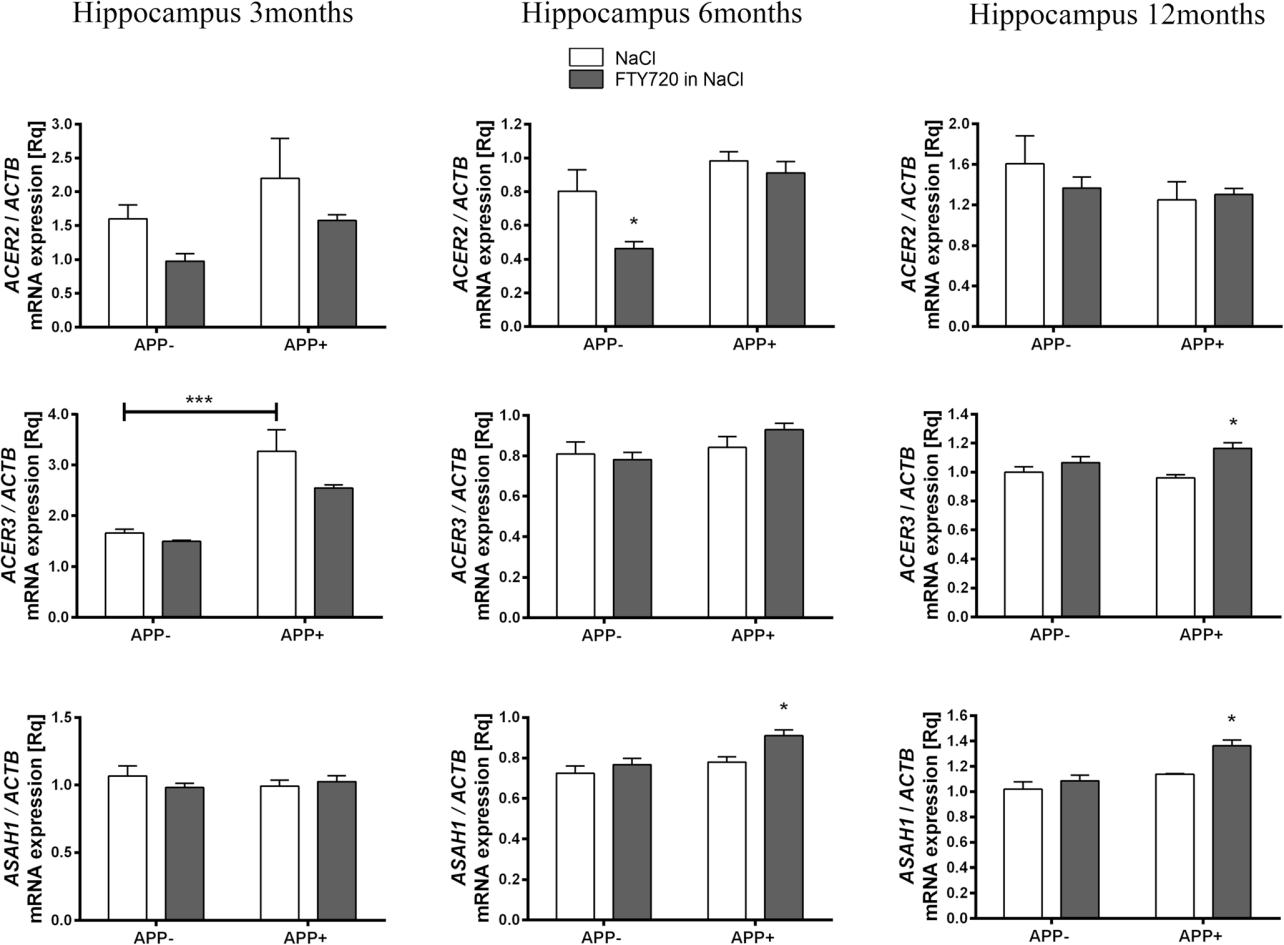
The influence of fingolimod on the gene expression of ceramidases in the hippocampus of *AβPP* (V717I)-transgenic mouse. mRNA expression of *ACER2*, *ACER3*, and *ASAH1* was measured with real-time PCR in the hippocampus of *AβPP*-transgenic and control mice at the age of 3, 6, and 12 months. *p < 0.05; ***p < 0.001 as compared with the corresponding controls (horizontal bar: APP^−^ vs. APP^+^; stars without the bar: the effect of FTY720 treatment within each group)—ANOVA with Tukey post hoc test

Ceramidases convert ceramide further into sphingosine on the *salvage pathway*; sphingosine is considered as a pro-apoptotic compound, and may also serve as a precursor for the strongly anti-apoptotic S1P. We observed higher mRNA for *ACER3* in 3-month-old hippocampus of APP^+^ mice as compared with APP^−^ animals (Fig. 3). Together with the above-mentioned changes in ceramide synthases, it suggests not only intensified ceramide generation but also its utilization, leading to accelerated metabolic turnover between ceramide and sphingosine. However, no changes in ceramidases were observed at 6 or 12 months of age (Fig. 3).

**Fig. 4:**
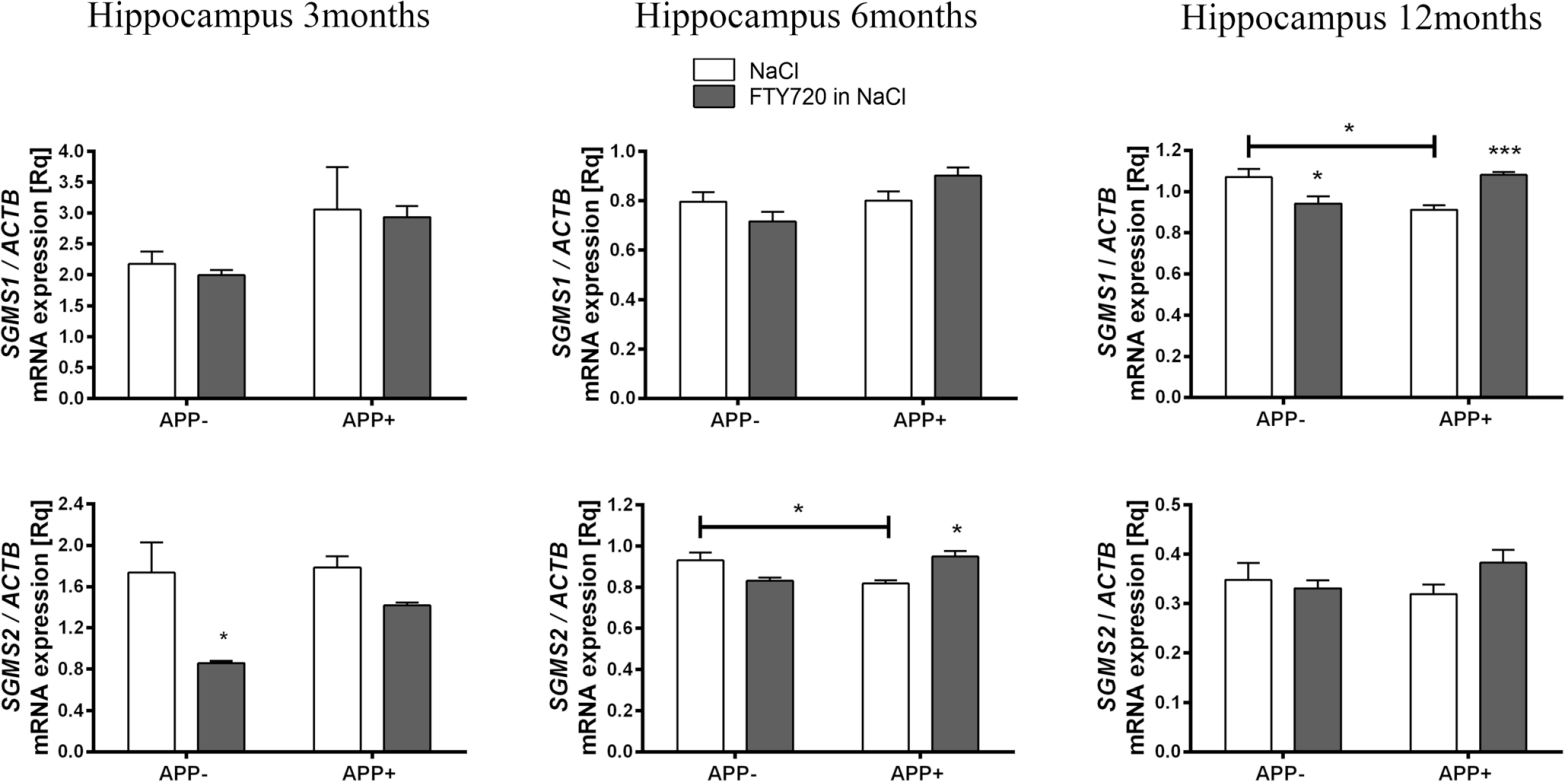
Fingolimod-induced changes in the gene expression of sphingomyelin synthases in the hippocampus of *AβPP* (V717I)-transgenic mouse. mRNA expression of *SGMS1* and *SGMS2* was measured with real-time PCR in the hippocampus of *AβPP*-transgenic and control mice at the age of 3, 6, and 12 months. *p < 0.05; ***p < 0.001 as compared with the corresponding controls (horizontal bar: APP^−^ vs. APP^+^; stars without the bar: the effect of FTY720 treatment within each group)—ANOVA with Tukey post hoc test

**Fig. 5:**
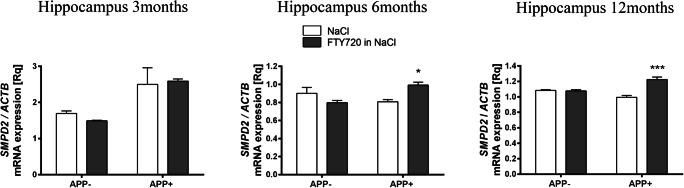
The influence of fingolimod on mRNA expression of sphingomyelinase in the hippocampus of *AβPP* (V717I)-transgenic mouse. mRNA expression of *SMPD2* was measured with real-time PCR in the hippocampus of *AβPP*-transgenic and control mice at the age of 3, 6, and 12 months. *p < 0.05; ***p < 0.001 as compared with the corresponding controls—ANOVA with Tukey post hoc test

Ceramide is also utilized on the *sphingomyelinase pathway* by sphingomyelin synthases (SGMS). Significant reduction was observed in APP^+^ hippocampus at 6 months (SGMS2) and 12 months (SGMS1), as shown in Fig. 4. The differences in subcellular localization and some functions of the two sphingomyelin synthases [63, 64] might affect the outcome of their age-specific regulation. The sphingomyelinase SMPD2, which catalyzes the opposite reaction, did not change significantly at any age, thus suggesting a shift from sphingomyelin towards ceramide (Fig. 5).

Fingolimod (FTY720) notably countered the AβPP-induced elevation of *CERS2* expression in 3-month-old APP^+^ hippocampus. At 6 months of age, no effect of FTY720 on ceramide synthases was observed. At 12 months, FTY720 only upregulated *CERS4*, which was unaffected by AβPP transgene (Fig. 2). Similarly, fingolimod also had age-dependent influence on the hippocampal expression of ceramidases, which remained unchanged by AβPP: it significantly increased *ASAH1* at 6 months and *ACER3* and *ASAH1* at 12 months (Fig. 3). Fingolimod also countered the Aβ/AβPP-related drop of sphingomyelin synthases: *SGMS2* at 6-month-old and *SGMS1* at 12-month-old hippocampus (Fig. 4). However, FTY720 also increased the expression of *SMPD2*, which was unchanged by AβPP (at 6 and 12 months—Fig. 5).

**Fig. 6:**
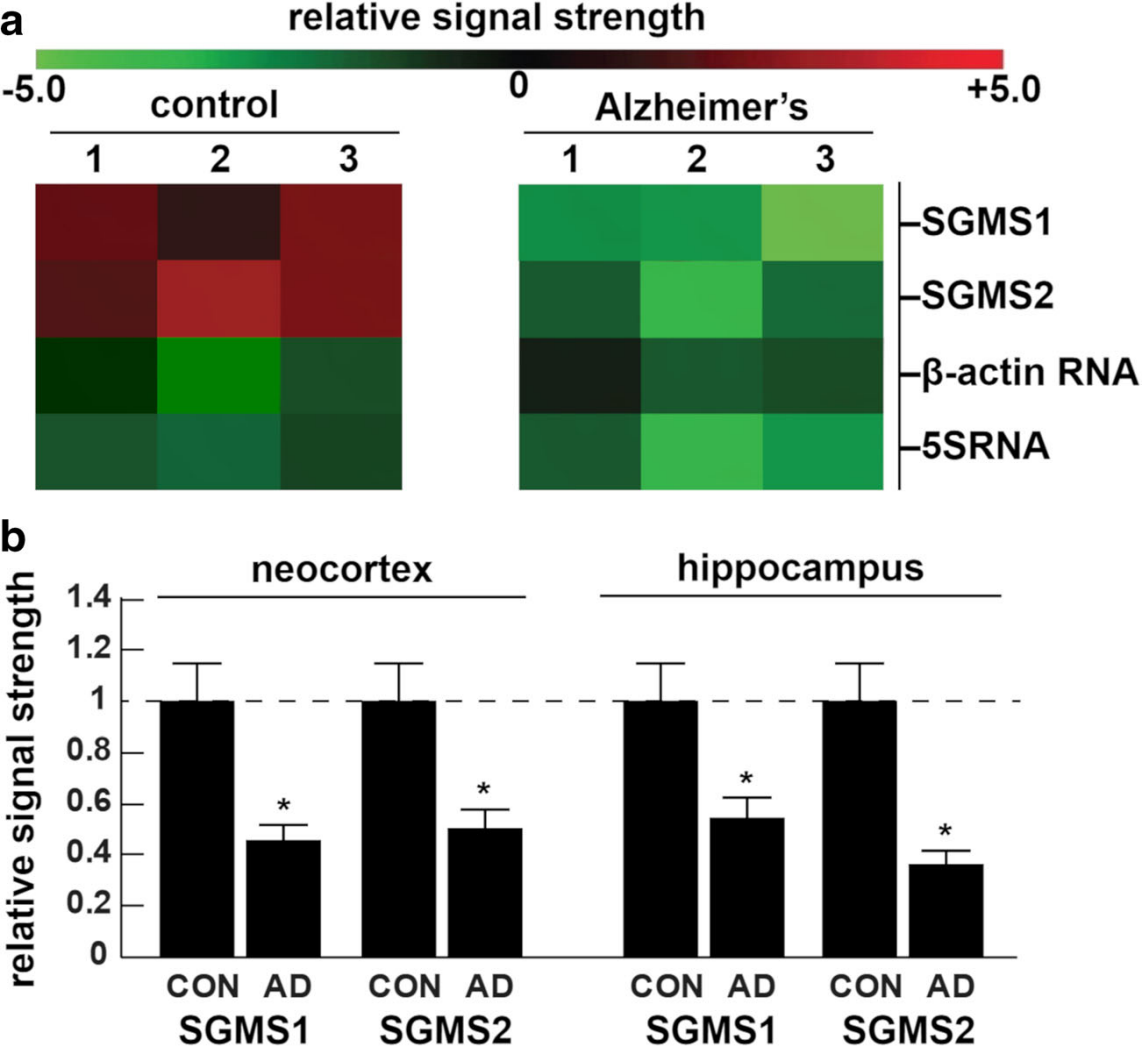
SGMS1 and SGMS2 mRNA deficits in AD neocortex and hippocampus when compared with age-matched controls. **a** Brain tissue hippocampal samples (*n* = 3) were analyzed for mRNA abundance from control (*n* = 3) and AD brains (*n* = 3); control mean age ± one standard deviation was 70.3 ± 8.5 years; and AD mean age ± one standard deviation was 71.7 ± 9.2 years; there were no significant differences in the age, total RNA yield, post-mortem interval (all PMI ~ 3 h or less), or total RNA purity between the control and AD samples; the control markers β-actin and 5S RNA showed no difference in abundance between control and AD; however, in the hippocampus, SGMS1 and SGMS2 were reduced to 0.54 and 0.36 of age-matched neurologically normal control values, respectively. **b** Data in bar graph format for the levels of SGMS1 and SGMS2 mRNA in AD neocortex and hippocampus; a dashed horizontal line at 1.0 is included for ease of comparison and represents control signals in each of the 4 determinations; for both neocortex and hippocampus (*n* = 3); *p < 0.05 (ANOVA)

Control- and age-matched human neocortex and hippocampus samples were also analyzed for mRNA abundance for the sphingomyelin synthases *SGMS1* and *SGMS2*. Both *SGMS1* and *SGMS2* were observed to be downregulated in both AD neocortex and hippocampus when compared with age-matched controls (Fig. 6). The levels of mRNA for CERS, ACER, ASAH, and SMPD were not significantly altered between control and AD samples (data not shown).

## Discussion

Evidence is accumulating for the engagement of sphingolipid signaling in the pathogenesis of AD. The alterations that favor ceramide signaling over S1P occur very early in the disease development and correlate with its severity [6, 8]. Ceramide generation is enhanced during what has been proposed as pre-MCI (pre-*mild cognitive impairment*), and diminishes at late disease stages [7]. The AD-linked changes appear to be an escalation of trends observed in healthy aging [5], suggesting that sphingolipids might be a part of the mechanisms that link AD with advanced age. The sources of the age-/AD-related disturbances in the metabolism of bioactive sphingolipids are not fully understood. The ability of cell surface S1P receptors to regulate gene expression via AP-1 and NF-κB is a very important aspect both due to its potential significance for the disease mechanism, and to its usefulness as a research (and potentially therapeutic) tool.

The murine AβPP (V717I)-transfected model has been shown to largely replicate an array of AD features in a characteristic time sequence [49, 50] (see “Materials and Methods”), including numerous changes in sphingolipid metabolism/signaling observed in the diseased brain [27]. Together with the results from cellular models [13], it suggests that the observed disturbances in sphingolipids might stem in a relatively direct way from the influence of Aβ, further stressing the possible critical role of sphingolipids in the core mechanisms of Aβ neurotoxicity. In our previous study, we observed age- and AβPP (V717I)-related reduction of *SPHK2*, *CERK*, and *S1PR*s mRNAs, which largely mirrored the changes observed in human hippocampal post-mortem AD material (lower *SPHK1* and *SPHK2*, *S1PR1*, and *CERK*) [27]. The reduction of sphingosine kinase expression should have negative impact upon cellular survival, and indeed, *BCL2* levels were reduced [27].

While the *sphingolipid rheostat* model originally ascribed clearly distinct pro- or anti-apoptotic roles to respectively ceramide and S1P/C1P [9], newer data suggest a more complex picture [65, 66]. However, our previous results on the age-related reduction of *BCL2* expression in AβPP-transgenic mice seem to confirm that in the case of Aβ neurotoxicity there is a shift towards elevated pro-apoptotic signaling at the expense of the survival signals [27]. It is also likely that changes in the multiple signaling and neurotransmitter pathways that interact with sphingolipids [14] impact neuronal phenotype (synaptic maintenance and function, regulation of mitochondrial hemeostasis, stress response). In the current work, we have noted that in the absence of changes in the rate-limiting serine palmitoyltransferase, upregulation of three ceramide synthases in the 3-month-old hippocampus (elevated *CERS6* persisted into the age of 6 months), together with one of the ceramidases (*ACER3*), may alter the metabolic turnover of ceramide. However, the changes seem to occur at an early age, long before the disease symptoms fully develop in the model. It corresponds to a degree with the clinical data suggesting early, pre-MCI spike of ceramide production [7]. It has recently been proposed that hippocampal changes in ceramide synthases could also be involved in alterations of oxidative stress, mitochondrial respiration, energy and fatty acid metabolism, transcriptional regulation, and DNA repair [reviewed in 67–70]. Ceramides and other sphingolipids are known to influence activities of transcriptional regulators including p53, AP-1, NF-κB, signal transducer and activator of transcription 3 (STAT3), or the splicing regulator serine/arginine-rich splicing-factor 1 (SRSF1) [71–73; reviewed in 14 and 67]. Sphingolipid metabolism is also implicated in DNA damage response regulating proteins such as poly(ADP-ribose) polymerase, p21, p53, or retinoblastoma (Rb) [reviewed in 74 and 75].

**Fig. 7:**
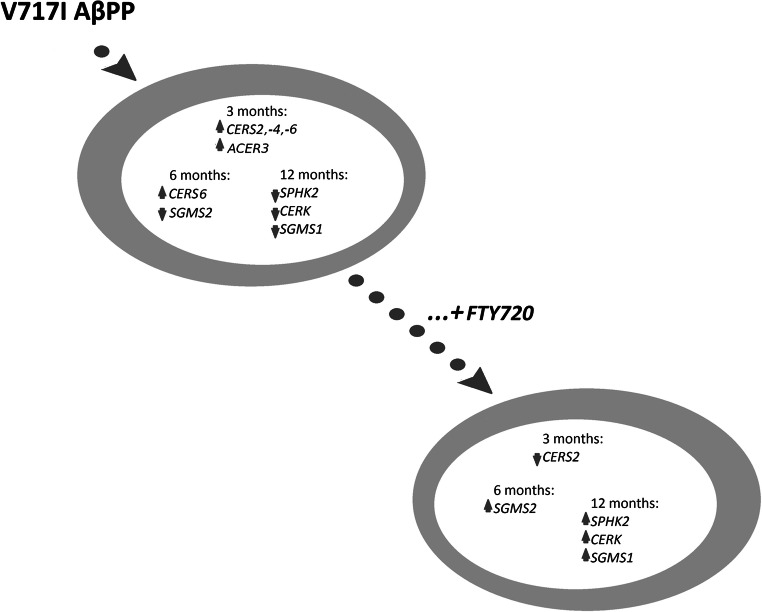
The influence of fingolimod (FTY720) on Aβ-/AβPP-altered sphingolipid metabolic enzymes. Female FVB-Tg(Thy1; APP LD2/B6) mice expressing human AβPP with the “London” V717I mutation display altered expression of sphingosine kinase *SPHK2*, ceramide kinase (*CERK*), and sphingomyelin synthases (*SGMS1*, *SGMS2*) in an age-dependent manner (different sets of changes at 3, 6, and 12 months of age ([27] and current results). Treatment of AβPP-transgenic animals with FTY720 reverses these changes (in addition to triggering further alterations in mRNAs which do not respond to Aβ/AβPP—omitted for clarity)

Fingolimod is a clinically available modulator of S1PR-dependent signaling events, including gene transcription. Strikingly, its effects on AβPP-expressing brains appear different from those on control tissue, suggesting that it might interact with the mechanisms of Aβ/AβPP impact. Reduced mRNA for *S1PR1* and upregulated *S1PR3* we previously observed in AD brains might lead to changes in the G protein–mediated regulation of genes engaged in sphingolipid metabolism and signaling [10, 11, 27]), Thus, altered *S1PR1* may change fingolimod’s effects in the Alzheimer’s brain. Indeed, in the current work, we have observed widespread modification of the gene expression pattern in fingolimod-treated APP^+^ mice in an age-dependent manner (Fig. 7). *SPHKs* and *CERKs* responded positively to the treatment of 12-month-old APP^+^ animals [27]. However, our current results demonstrate a shift in the reaction of sphingolipid metabolism to fingolimod administration in the hippocampus, occurring between 6 and 12 months of age. Importantly, at 3 months, FTY720 reduced the mRNAs for ceramide synthase 2, potentially blunting the already accumulating pro-apoptotic stimulus. A similar trend appeared to affect *CERS4* and *CERS6*, although without reaching significance. At 6 months, fingolimod in turn upregulated ceramidase *ASAH1* and increased both enzymes of the ceramide—sphingomyelin metabolism (*SGMS2*, *SMPD2*), which may have impact not only on ceramide but also on other signaling pathways (such as diacylglycerol- or lipid raft-dependent ones). At 12 months, the compound increased *SGMS1*, which was reduced in the AβPP-transgenic animals, and also increased several genes (*CERS4*, *ACER3*, *ASAH1*, and *SMPD2*), which remained at the same levels in transgenic and transgene-lacking control animals. The corresponding proteins take part in both the production (CERS4, SMPD2) and the utilization of ceramide (ACER3, ASAH1). These effects may be highly important aspects of the proposed neuroprotective activity of fingolimod in AD, suggesting its influence in the very early stages of the neurodegenerative processes (Fig. 7) in addition to the previously published restoration of *BCL2* expression at later stages. Our results also confirm the reduction in the brain-enriched sphingomyelin synthases *SGMS1* and *SGMS2* also in human AD neocortex and hippocampus (Fig. 6), suggesting a deficiency in the capability to synthesize sufficient amounts of sphingomyelin in anatomical regions of the brain targeted by the AD process.

Thus far, fingolimod’s effects on AD and its models were largely analyzed from the perspective of the established immunomodulatory function of sphingolipid signaling [76]. Aβ is considered to trigger immune activation of glial cells, which attempt to remove it from extracellular space, but can accelerate the damage to neighboring neurons as a side effect. Fingolimod has been shown to reduce inflammatory markers in CA1/subiculum and the numbers of activated microglial cells in the hippocampus in the 5×FAD mouse model. In the same time, fingolimod reduced Aβ_42_ plaque density and the total level of Aβ_42_ [77]. The mechanism of Aβ accumulation may be augmented by proinflammatory environment in the diseased brain, which is suggested to impair Aβ clearance by astrocytes [78]. Mice overexpressing AβPP and presenilin 1 display activated local astroglia and microglia, infiltration of peripheral macrophages and natural killer cells [79]. Infections, susceptibility to which rises in old age, are noted to accelerate the cognitive deterioration of AD patients [80] and to increase Aβ burden in animal models [78]. FTY720 has been shown to inhibit the infection-related activation of astrocytes, and to prevent accumulation of soluble and plaque Aβ in the mouse brain probably through stimulation of Aβ phagocytosis [78]. Fingolimod also restores neurotrophin production and blocks neuronal death occurring in response to Aβ [34, 81]. These effects lead to improved associative learning and object recognition in mice receiving stereotactic injections of oligomeric Aβ [81]. FTY720 has also been tested in models of other neurodegenerative disorders and provided Akt-mediated neuroprotection in a mouse model of Parkinson’s disease [82]. These results only show some aspects of the usefulness of fingolimod as a research tool and potential treatment in neurodegeneration. However, the wide spectrum of interactions of genetic background, age, and environmental factors with sphingolipids, signaling pathways such as PI3K-Akt, Aβ/AβPP metabolism, and transcription factors/histone deacetylases pose a great challenge in pharmacotherapy. The increasing complexity of sphingolipid-based modulation of neuronal phenotype and survival in the diseased brain and its reactions to treatment [27] urges for further elucidation of the multiple effects of fingolimod/FTY720 in Alzheimer’s disease.

## Concluding Remarks

Our result confirms that the presence of disease-associated (V717I-mutated) Aβ precursor protein leads to early changes in the expression of sphingolipid-related genes, mainly those engaged in ceramide biosynthesis. During the process of aging, the pattern of hippocampal expression evolves in likely interaction with the ongoing pathological changes. Enhanced expression of ceramide synthases and ceramidase starting from 3 months of age suggests intensification of sphingolipid metabolism in early stages of the disease. Increased ceramide synthase persisted into the age of 6 months; at the same age, reduction of sphingomyelin synthase appeared in APP^+^ hippocampus (*SGMS2*) and the trend continued into the age of 12 months (*SGMS1*). Thus, at 6 and 12 months, the AβPP-induced changes in gene regulation may lead to the observed imbalance between ceramide and S1P (sphingosine-1-phosphate) in the direction of ceramide signaling, triggering crucial deregulation in survival/death equilibrium.

Sphingosine-1-phosphate receptor modulator FTY720 (fingolimod) counters the observed Aβ-/AβPP-evoked disturbances in a spatiotemporally specific fashion, showing its potential to alter the disease course. We have previously confirmed apparent pro-survival reaction to fingolimod in the hippocampus of 12-month-old AD model mice that involved sphingosine kinase, ceramide kinase, and *BCL2* [27]. However, our current results suggest much more complex image (Fig. 7). While the changes at 3 months seem to favor reduction of ceramide levels, at 12 months, fingolimod accelerated metabolic turnover of ceramide also in the *sphingomyelinase pathway* and induced further alterations unrelated to the effects of AβPP. These novel findings prompt for both caution and hope in the consideration of fingolimod as a candidate repurposed drug for the treatment of AD and other neurodegenerative disorders. Further extensive examination of fingolimod’s effects is necessary.
